# Development and evaluation of an online surgical elective for medical students

**DOI:** 10.1186/s12909-023-04180-w

**Published:** 2023-04-17

**Authors:** Mary Goble, Neil Chapman

**Affiliations:** 1 Academic Unit of Medical Education, Sheffield, UK; 2 Department of Oncology and Metabolism, The Medical School, The University of Sheffield, Beech Hill Road, Sheffield, S10 2RX UK; 3 Faculty of Medicine, Imperial College London, Exhibition Road, London, SW7 2BX UK

**Keywords:** Surgical Education, Online learning, Medical undergraduate curriculum, Surgical Elective, Virtual elective surgery, E-Learning

## Abstract

**Background:**

Decreased experiential learning opportunities exacerbated by the COVID-19 pandemic have increased development of online surgical educational courses. To what extent may such courses provide exposure to broad and accessible surgical education?

**Methods:**

Surge is a 6-week online surgical elective hosted within a virtual learning environment, covering all surgical specialties. Course content is mapped to the Royal College of Surgeons’ Undergraduate Curriculum in Surgery. Each week consultant surgeons discuss their specialty in short videos on anatomy, pathology and lifestyle of a surgeon. Students also engage with learning activities; further reading lists; formative quizzes and live sessions including suturing practice. Participants were medical students undertaking third-year electives at the University of Sheffield. Pre- and post-course questionnaires investigated student interest in surgery, understanding of steps required to pursue a surgical career and confidence in surgical environments. Qualitative data was collected via free-text responses and analysed with content analysis. Quantitative data was collected using 5-point Likert scales (1 = Strongly Disagree; 5 = Strongly Agree) and analysed using the Wilcoxon signed-rank test.

**Results:**

Twenty-two students participated in Surge over five 6-week cohorts. Examination of free-text responses revealed students gained increased understanding of available surgical career options. Students felt better informed regarding different surgical specialties (median score 2.5 vs. 4, *p* = 0.000) and steps required to develop a surgical portfolio (median score 2 vs. 5, *p* = 0.000). Additionally, confidence in understanding of relevant intraoperative steps improved (median score 3 vs. 4, *p* = 0.000).

**Conclusion:**

These data demonstrate Surge increased student confidence and understanding of surgical careers despite reduced in-person opportunities to engage with surgical education. Surge will continue to be developed and evaluated on a larger scale.

**Supplementary Information:**

The online version contains supplementary material available at 10.1186/s12909-023-04180-w.

## Introduction

Historically, many students have been deterred from considering a surgical career due to factors including poor work-life balance [1], however interventions at undergraduate level, such as career days, conferences, and short courses may reverse these preconceptions and have a positive impact on student interest [2].

Cancellation of elective surgical procedures as a result of the Covid-19 pandemic across the world led to a reduction of medical student exposure to surgical training [3–5]. This affected not only their clinical learning, but also their ability to practise skills and develop a career interest in surgical specialties [4, 5]. To compensate for this loss, medical schools witnessed an increased utilisation of online educational platforms [6].

Different specialties, such as plastic surgery [7], ophthalmology [8] and general surgery [9] developed virtual surgical courses to bridge the gap between student interest in pursuing surgical placements and low availability of these placements. Students were exposed to common procedures, seminars and grand rounds via online content and live access to hospital meetings broadcasted online. These courses were well perceived by students; indeed, Grady et al. [9] report an increased understanding of interest and knowledge in general surgery. Online learning software and virtual platforms more generally have seen an exponential increase in use and popularity and have been demonstrated to be valuable additions to the surgical training toolbox [10].

Surge is an online elective providing a comprehensive overview of surgical specialties at a level appropriate for medical undergraduates. It was developed in response to the COVID-19 pandemic with the aims of increasing exposure to surgery, improving understanding of surgical career paths available, and providing the opportunity for students to develop their practical skills. As such, this study aimed to address the following question: To what extent does Surge provide exposure to broad and accessible surgical education?

## Methods

The online course was designed to fit a six-week long student-selected component or elective at a UK university. It was hosted on Moodle, an online server widely used for massive open online courses (MOOCs).

The course content was developed using the *Undergraduate Curriculum for Surgery* by the Royal College of Surgeons of England [11]. Consultant surgeons in each specialty were approached and given the opportunity to give short talks based on the learning objectives defined by the Undergraduate Curriculum and their experience of appropriate teaching goals. Third-year medical students were consulted regarding the course design and content; based on their feedback topics were separated into focussed 15–30-minute videos to maintain engagement during the course.

**Fig. 1:**
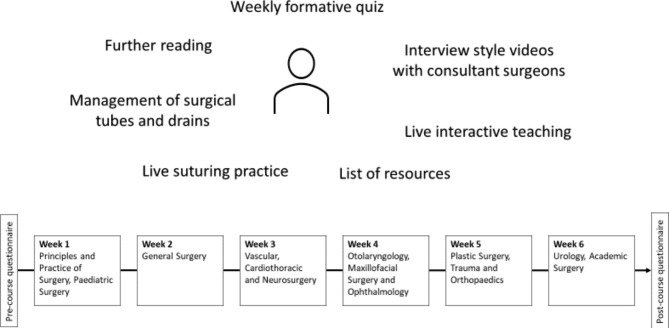
Content and structure of the course

The course content and structure is illustrated in Fig. 1. Each of the six weeks covered two to three surgical specialties. For each specialty, a consultant or senior registrar delivered between two and six talks on anatomy, common pathology, and procedures. They also recorded an interview style video with a medical student, answering lifestyle questions and career advice. 50% (7/14) of the clinicians delivering teaching were women. Each week interactive lessons were provided in a written format on topics such as peri-operative care and the history of surgery, as well as a formative quiz, and a list of resources for further reading. Certain sessions were delivered live via Googlemeet to complement recorded teaching, helping build familiarity between course participants and leads. Topics covered included the theatre environment, critical appraisal, and getting involved in research as a medical student. Students were provided with basic suturing kits and sutures to enable virtually delivered live suturing teaching. A surgical registrar with a camera set-up enabling two angles of vision of their hands gave small group tutoring sessions online, in which they covered instrument handling, hand and instrument ties, and continuous and interrupted sutures. Students were able to demonstrate their skills and receive live feedback via camera; they used integrated cameras on their personal computers or laptops, or used their mobile phones to allow the tutor to visualise their hands.

Feedback was collected each week regarding the quality of the videos, teachers, quiz, live teaching sessions, and resources on a 5-point Likert scale (Appendix 1). Students had the opportunity to give free-text feedback. They completed a pre-course and post-course questionnaire which investigated their interest and exposure to surgery prior to and after completing the course on a 5-point Likert scale (1 = Strongly Disagree, 5 = Strongly Agree), and in free text boxes (Appendix 2).

Retention of content taught on the course was not tested for the initial few cohorts, however it became clear it would be prudent to collect such data. Subsequently, for the last cohort, retention of the course content was measured by repeating quiz questions at the end of the six weeks.

Ethical approval was obtained from the University of Sheffield Ethics Committee. Data was anonymised and stored securely following data protection policies of the University of Sheffield. All methods were carried out in accordance with the relevant University guidelines and regulations.

Quantitative data was analysed using IMB SPSS version 26 [12], it was tested for normality and analysed using non-parametric paired sample analysis (Wilcoxon Rank Signed test). Statistical significance was taken as p < 0.05. Qualitative data was manually coded on Excel [13] using thematic content analysis.

## Results

Twenty-six students completed the course in total, they were split into five different cohorts (Cohort 1 = 3 students, cohort 2 = 4 students, cohort 3 = 4 students, cohort 4 = 9 students, cohort 5 = 6 students). Each cohort completed the course in six weeks. Participants were medical students in a UK university who applied to the course for their third-year elective. Four students did not complete the post-course questionnaire therefore were excluded from analysis, sample size n = 22.

1. Qualitative

Evidence suggests medical school curricula do not always provide the opportunity for clinical placements in each surgical specialty, resulting in students feeling like they would benefit from increased teaching and career exposure to specific specialties [14, 15]. Content analysis of the free-text feedback corroborated these previous findings, revealing that students had joined the course for reasons such as “[to gain] an all-round better understanding”, to “consider […] surgery as a future career option”, and for the “opportunity to gain experience in a wide variety of surgical specialties”. This stemmed from a feeling of “[having] not received enough experience or teaching about surgery” and “little exposure to surgery”. They wanted to gain an “insight into surgery as a whole”, “clarify myths/stereotypes”, and “build a portfolio”.

Analysis of free-text responses post-course revealed that students gained an understanding of “how wide and varied the field of surgery is”, and that it is “massively diverse”. Students also reported they “understood the application process”, felt that “surgery [was] not as daunting due to the teaching provided”, and that the course “will allow [them] to make informed decisions about [their] career planning”. In addition, students found personal interviews with consultants a highlight of the course and felt they “were having a 1-on-1 chat with the respective surgeons”; “their answers were very personal which gave a good insight into the career”.

2. Quantitative

Results of the pre- and post-course questionnaire were tested for normality. Data was not normally distributed therefore was analysed using Wilcoxon Signed Rank test. Data is displayed in Table 1.

**Table 1:** Pre- and post-course Questionnaire Results

Question 1 = Strongly Disagree 2 = Disagree 3 = Neither Agree nor Disagree 4 = Agree 5 = Strongly Agree	Pre-course (median) n = 22	Post-course (median) n = 22	Z score, p value
I am interested in pursuing a surgical career	3.5	4	Z=-2.18, p = 0.03
I have been given the opportunity to explore my career interests through medical school	3	4	Z=-1.94, p = 0.05
I have been to a surgical theatre	5	5	Z=-0.81, p = 0.41
I have had the opportunity to be involved in research on a surgical topic	2	2	Z=-1.76, p = 0.08
I have had the opportunity to attend surgical courses and/or conferences	3	3.5	Z=-2.99, p = 0.003
I have been able to attend surgical talks/ lectures (including online talks) outside of mandatory education by the medical school	4	4	Z=-0.72, p = 0.47
I am confident in scrubbing up in theatre	4	4	Z=-2.30, p = 0.02
I am confident in assisting in theatre	2.5	3	Z=-1.67, p = 0.09
I am confident in speaking to surgeons	3	4	Z=-3.23, p = 0.001
I am confident in understanding what is happening during an operation	3	4	Z=-3.75, p = 0.000
I have considered a career in surgery	4	4	Z = 0.33, p = 0.739
I feel well informed regarding the different specialties which exist in surgery	2.5	4	Z=-3.87, p = 0.000
I feel well informed regarding the steps I can take as a medical student to develop a surgical portfolio	2	5	Z=-3.99, p = 0.000
I believe online learning is a useful tool to supplement learning at medical school	4	4	Z=-2.673, p = 0.008
Online learning courses are suitable way to develop my interest in surgery	4	4	Z=-0.447, p = 0.655

After the course, students were more interested in pursuing a surgical career (median score 3.5 vs. 4, Z=-2.18, p = 0.03), felt they had had more opportunities to explore their career interest (median score 3 vs. 4, Z=-1.94, p = 0.05), and more opportunities to attend surgical courses and conferences (median score 3 vs. 3.5, Z=-2.99, p = 0.003).

Student confidence in the surgical environment also increased: they felt more confident speaking with surgeons (median score 3 vs. 4, Z=-3.23, p = 0.001), and understanding the progression of an operation (median score 3 vs. 4, Z=-3.75, p = 0.000).

There was no change in how much they had been to theatre (median score 5 vs. 5, Z=-0.81, p = 0.41), their confidence in scrubbing (median score 4 vs. 4, Z=-2.30, p = 0.02), their perceived opportunities to be involved in surgical research (median score 2 vs. 2, Z=-1.76, p = 0.08) or attend surgical talks outside of mandatory education (median score 4 vs. 4, Z=-0.72, p = 0.47). They did not feel more confident assisting (median score 2.5 vs. 3, Z=-1.67, p = 0.09). There was no change to their considering a career in surgery (median score 4 vs. 4, Z = 0.33, p = 0.739).

Students felt better informed regarding different the surgical specialties (median score 2.5 vs. 4, Z=-3.87, p = 0.000) and the necessary steps to develop a surgical portfolio (median score 2 vs. 5, Z=-3.99, p = 0.000).

After the course, students were equally likely to believe online learning was useful to develop an interest in surgery (median score 4 vs. 4, Z=-2.673, p = 0.008, and there was no change to their opinion of online learning to supplement traditional learning in medical school as students continued agreeing it is useful (median score 4 vs. 4, Z=-0.447, p = 0.655).

Six students completed a post-course quiz which repeated all formative questions contained in the course. Their median score improved on repeating the questions (median score 7.3 vs. 8.9, Z=-2.20, p = 0.028).

All students completed the entirety of the course.

## Discussion

Mentorship is of recognised importance in the field of surgery due to the practical nature of the specialty, requiring personalised training, and the demanding nature of the field: mentorship encompasses technical learning as well as career support and advice [16–18]. Whilst the value of mentors is undeniable, students at times find the process of finding an appropriate mentor daunting; Entezami et al. also report on the lack of female mentors [16, 19]. Mentorship was offered in this online course via personable interviews with a variety of surgeons, of which half were women, which helped students understand surgery is a diverse field which offers many career paths to students, as indicated by free-text answers which reveal that students joined the course to develop their understanding of surgical specialties and dispel possible myths surrounding surgical careers. Students felt much of the added value of the course was gained by watching 1–1 interview-style videos with consultants or senior registrars, which seemingly made them more approachable thereby allowing students to envision themselves pursuing similar careers. This is corroborated by the results of the pre- and post-course questionnaire, which suggest that a major effect of the course was that students agreed to feeling better informed regarding surgical specialties (median score 2.5 vs. 4, Z=-3.87, p = 0.000). They strongly agreed that they understood how to build a surgical portfolio (median score 2 vs. 5, Z=-3.99, p = 0.000). The course provides a comprehensive overview of all surgical specialties and has the advantage of including one-on-one interviews with surgeons in each specialty, providing insight which can only be gained via personal interactions rather than textbook learning. Whilst the recorded nature of the course does not offer the same degree of individuality as real-life mentorship, Surge may be an interesting addition to traditional mentorship relationships. Virtual mentorship is an avenue to be explored to democratise the mentor-finding process and offer increased opportunities for students to engage with mentors of the same gender, ethnicity, and socio-economic background [20, 21].

By completing the course students felt they had more opportunities to attend surgical courses and conferences; Surge directed students towards resources such as professional organisations which organise undergraduate taster days, or societies which host conferences. Surge highlights the existence of such events to students and may encourage them to seek out similar opportunities for themselves once introduced to the types of setting in which they are typically found.

Lyon describes the experience of students in the operating theatre, identifying the need for adequate student preparation and orientation in order for them to benefit the most from this learning experience [22]. Through this online course, students learn the principles of gloving and gowning, are familiarized with the surgical equipment and common surgical procedures, helping them develop “noticing skills” prior to entering the operating theatre. Results demonstrate their confidence in the surgical environment increased after the course. This is an encouraging finding which suggests students nationally and internationally may benefit from an orientation-style course similar to the one described in this paper in order to potentiate learning in the surgical environment.

Despite the opportunity to practice their suturing skills, students did not highlight this as a particularly valuable addition to the course. There was no significant improvement in their confidence in assisting in theatres. MacGann et al. [23] in their evaluation of a week-long online suturing elective highlight that students do find virtual teaching of technical skills to be useful and they value in self-directed practice time in particular. This suggests an area for development of this course, which may be achieved by increasing time dedicated to technical skills. In the subset of students who were tested for retention of content taught on the course, scores increased when questions were repeated on completion on the course, indicating improved knowledge on these topics. Despite the sample size of students being limited, these results encourage further data collection and analysis regarding content retention in the course.

Overall Surge was well received by medical students and can be used to supplement learning and develop interest in surgical careers despite reduced clinical exposure to the theatre environment. Students completed the course entirely, which indicates they were engaged with the content throughout; however, they had chosen to participate over various options offered to them by the Medical School, which may bias towards higher than average motivation to complete the course. It has the scope to be developed and assessed further.

Limitations of this study were small number of participants, which limited the generalisability of study findings. This may have also facilitated the delivery of the live components of the course, whilst a larger cohort of students may require breakout rooms and an increased number of facilitators to comfortably deliver of live sessions and ensure high levels of participation.

## Conclusion

Surge is a six-week online surgical elective which provides exposure to broad and accessible surgical education, increasing student understanding of surgical career paths and development of surgical portfolios. Students felt online learning was a useful way to approach surgical topics and, in particular, found value in personal interviews with surgeons. Surge has the potential to complement traditional mentorship structures and enhance student experience in the operating theatre. It is an easily accessible course which can be offered to students internationally; this type of learning experience should be developed and evaluated further.

## Electronic supplementary material

Below is the link to the electronic supplementary material.

Supplementary Material 1

Supplementary Material 2

## Data Availability

The datasets used and/or analysed during the current study are available from the corresponding author on reasonable request.
