# Supplementary material for: Development and evaluation of an online surgical elective for medical students
Source: BMC Med Educ. 2023 Apr 17;23:254. doi: 10.1186/s12909-023-04180-w (PMC10108795; doi:10.1186/s12909-023-04180-w)
Supplement: Supplementary file 1 — Supplementary Material 1 [file 12909_2023_4180_MOESM1_ESM.docx]

**Pre-course questionnaire**

Why have you chosen to take part in this course?

| Free text |
| --- |

What do you hope to gain from this course?

| Free text |
| --- |

I am interested in pursuing a surgical career

| Strongly agree | Agree | Neither agree nor disagree | Disagree | Strongly disagree |
| --- | --- | --- | --- | --- |

| Cardiothoracics | Plastics | MaxFax | Paediatric | Vascular | ENT | Neurosurgery | Urology | General | Trauma and orthopaedics |
| --- | --- | --- | --- | --- | --- | --- | --- | --- | --- |

I am interested in the following surgical specialties.

I have been given the opportunity to explore my career interests through medical school

| Strongly agree | Agree | Neither agree nor disagree | Disagree | Strongly disagree |
| --- | --- | --- | --- | --- |

I have been to a surgical theatre

| Strongly agree | Agree | Neither agree nor disagree | Disagree | Strongly disagree |
| --- | --- | --- | --- | --- |

I have had the opportunity to be involved in research on a surgical topic

| Strongly agree | Agree | Neither agree nor disagree | Disagree | Strongly disagree |
| --- | --- | --- | --- | --- |

I have had the opportunity to attend surgical courses and/or conferences

| Strongly agree | Agree | Neither agree nor disagree | Disagree | Strongly disagree |
| --- | --- | --- | --- | --- |

I have been able to attend surgical talks/ lectures (including online talks) outside of mandatory education by the medical school

| Strongly agree | Agree | Neither agree nor disagree | Disagree | Strongly disagree |
| --- | --- | --- | --- | --- |

I am confident in scrubbing up in theatre

| Strongly agree | Agree | Neither agree nor disagree | Disagree | Strongly disagree |
| --- | --- | --- | --- | --- |

I am confident in assisting in theatre

| Strongly agree | Agree | Neither agree nor disagree | Disagree | Strongly disagree |
| --- | --- | --- | --- | --- |

I am confident in speaking to surgeons

| Strongly agree | Agree | Neither agree nor disagree | Disagree | Strongly disagree |
| --- | --- | --- | --- | --- |

I am confident in understanding what is happening during an operation

| Strongly agree | Agree | Neither agree nor disagree | Disagree | Strongly disagree |
| --- | --- | --- | --- | --- |

I have considered a career in surgery

| Strongly agree | Agree | Neither agree nor disagree | Disagree | Strongly disagree |
| --- | --- | --- | --- | --- |

I feel well informed regarding the different specialties which exist in surgery

| Strongly agree | Agree | Neither agree nor disagree | Disagree | Strongly disagree |
| --- | --- | --- | --- | --- |

I feel well informed regarding the steps I can take as a medical student to develop a surgical portfolio

| Strongly agree | Agree | Neither agree nor disagree | Disagree | Strongly disagree |
| --- | --- | --- | --- | --- |

Which area of training do you learn the most from?

| One-to-one | Small group tutorials | Lectures | Theatre | Ward round | Outpatient clinics |
| --- | --- | --- | --- | --- | --- |

I believe online learning is a useful tool to supplement learning at medical school

| Strongly agree | Agree | Neither agree nor disagree | Disagree | Strongly disagree |
| --- | --- | --- | --- | --- |

Online learning courses are suitable way to develop my interest in surgery

| Strongly agree | Agree | Neither agree nor disagree | Disagree | Strongly disagree |
| --- | --- | --- | --- | --- |

**Post-course questionnaire**

What was your main takeaway from this course?

| Free text |
| --- |

I am interested in pursuing a surgical career

| Strongly agree | Agree | Neither agree nor disagree | Disagree | Strongly disagree |
| --- | --- | --- | --- | --- |

| Cardiothoracics | Plastics | MaxFax | Paediatric | Vascular | ENT | Neurosurgery | Urology | General | Trauma and orthopaedics |
| --- | --- | --- | --- | --- | --- | --- | --- | --- | --- |

I am interested in the following surgical specialties.

I have been given the opportunity to explore my career interests through medical school

| Strongly agree | Agree | Neither agree nor disagree | Disagree | Strongly disagree |
| --- | --- | --- | --- | --- |

I have been to a surgical theatre

| Strongly agree | Agree | Neither agree nor disagree | Disagree | Strongly disagree |
| --- | --- | --- | --- | --- |

I have had the opportunity to be involved in research on a surgical topic

| Strongly agree | Agree | Neither agree nor disagree | Disagree | Strongly disagree |
| --- | --- | --- | --- | --- |

I have had the opportunity to attend surgical courses and/or conferences

| Strongly agree | Agree | Neither agree nor disagree | Disagree | Strongly disagree |
| --- | --- | --- | --- | --- |

I have been able to attend surgical talks/ lectures (including online talks) outside of mandatory education by the medical school

| Strongly agree | Agree | Neither agree nor disagree | Disagree | Strongly disagree |
| --- | --- | --- | --- | --- |

I am confident in scrubbing up in theatre

| Strongly agree | Agree | Neither agree nor disagree | Disagree | Strongly disagree |
| --- | --- | --- | --- | --- |

I am confident in assisting in theatre

| Strongly agree | Agree | Neither agree nor disagree | Disagree | Strongly disagree |
| --- | --- | --- | --- | --- |

I am confident in speaking to surgeons

| Strongly agree | Agree | Neither agree nor disagree | Disagree | Strongly disagree |
| --- | --- | --- | --- | --- |

I am confident in understanding what is happening during an operation

| Strongly agree | Agree | Neither agree nor disagree | Disagree | Strongly disagree |
| --- | --- | --- | --- | --- |

I have considered a career in surgery

| Strongly agree | Agree | Neither agree nor disagree | Disagree | Strongly disagree |
| --- | --- | --- | --- | --- |

I feel well informed regarding the different specialties which exist in surgery

| Strongly agree | Agree | Neither agree nor disagree | Disagree | Strongly disagree |
| --- | --- | --- | --- | --- |

I feel well informed regarding the steps I can take as a medical student to develop a surgical portfolio

| Strongly agree | Agree | Neither agree nor disagree | Disagree | Strongly disagree |
| --- | --- | --- | --- | --- |

Which area of training do you learn the most from?

| One-to-one | Small group tutorials | Lectures | Theatre | Ward round | Outpatient clinics |
| --- | --- | --- | --- | --- | --- |

I believe online learning is a useful tool to supplement learning at medical school

| Strongly agree | Agree | Neither agree nor disagree | Disagree | Strongly disagree |
| --- | --- | --- | --- | --- |

Online learning courses are suitable way to develop my interest in surgery

| Strongly agree | Agree | Neither agree nor disagree | Disagree | Strongly disagree |
| --- | --- | --- | --- | --- |

Please type in any other feedback you wish to leave in here

| Free text |
| --- |
