# Supplementary material for: Development and evaluation of an online surgical elective for medical students
Source: BMC Med Educ. 2023 Apr 17;23:254. doi: 10.1186/s12909-023-04180-w (PMC10108795; doi:10.1186/s12909-023-04180-w)
Supplement: Supplementary file 2 — Supplementary Material 2 [file 12909_2023_4180_MOESM2_ESM.docx]

**Appendix 1: Sample Feedback form**

Please tick as appropriate.

The content of the "Jaundice" video was relevant to my learning

| Strongly agree | Agree | Neither agree nor disagree | Disagree | Strongly disagree |
| --- | --- | --- | --- | --- |

| Strongly agree | Agree | Neither agree nor disagree | Disagree | Strongly disagree |
| --- | --- | --- | --- | --- |

The technical quality of the "Jaundice" video was adequate

| Strongly agree | Agree | Neither agree nor disagree | Disagree | Strongly disagree |
| --- | --- | --- | --- | --- |

The speaker Ms Sita Kotecha was knowledgeable

| Strongly agree | Agree | Neither agree nor disagree | Disagree | Strongly disagree |
| --- | --- | --- | --- | --- |

The "Post-operative Surgical Principles" activity was relevant to my learning

| Strongly agree | Agree | Neither agree nor disagree | Disagree | Strongly disagree |
| --- | --- | --- | --- | --- |

The format of the “Post-operative Surgical Principles" activity was adequate

| Strongly agree | Agree | Neither agree nor disagree | Disagree | Strongly disagree |
| --- | --- | --- | --- | --- |

The list of resources was useful

| Strongly agree | Agree | Neither agree nor disagree | Disagree | Strongly disagree |
| --- | --- | --- | --- | --- |

The week 2 formative quiz was useful

| Strongly agree | Agree | Neither agree nor disagree | Disagree | Strongly disagree |
| --- | --- | --- | --- | --- |

Please state at least one improvement which could be made to this week

| Free text answer |
| --- |

Please state at least one component of this week that you liked

| Free text answer |
| --- |
